# Applying LDA-based pattern recognition to predict isometric shoulder and elbow torque generation in individuals with chronic stroke with moderate to severe motor impairment

**DOI:** 10.1186/s12984-019-0504-1

**Published:** 2019-03-05

**Authors:** Joseph V. Kopke, Levi J. Hargrove, Michael D. Ellis

**Affiliations:** 1 Department of Biomedical Engineering, McCormick School of Engineering, 645 N Michigan Ave, Suite 1109, Chicago, IL 60611 USA; 2 Center for Bionic Medicine, 355 East Erie, Chicago, IL 60611 USA; 3 Department of Physical Therapy and Human Movement Sciences, Northwestern University, 645 N Michigan Ave, Suite 1100, Chicago, IL 60611 USA; 4 Department of Physical Medicine and Rehabilitation, Northwestern University, 710 North Lake Shore Drive, #1022, Chicago, IL 60611 USA

**Keywords:** Hemiparesis, Stroke, Flexion synergy, Shoulder, Pattern recognition, Linear discriminant analysis

## Abstract

**Background:**

Abnormal synergy is a major stroke-related movement impairment that presents as an unintentional contraction of muscles throughout a limb. The flexion synergy, consisting of involuntary flexion coupling of the paretic elbow, wrist, and fingers, is caused by and proportional to the amount of shoulder abduction effort and limits reaching function. A wearable exoskeleton capable of predicting movement intent could augment abduction effort and therefore reduce the negative effects of distal joint flexion synergy. However, predicting movement intent from abnormally-coupled torques or EMG signals and subsequent use as a control signal remains elusive. One control strategy that has proven viable, effective, and computationally efficient in myoelectric prostheses for use in individuals with amputation is linear discriminant analysis (LDA)-based pattern recognition. However, following stroke, shoulder effort has been shown to have a negative effect on classification accuracy of hand tasks due to the multi-joint torque coupling of abnormal synergy. This study focuses on the evaluation of an LDA-based classifier to predict individual degrees-of-freedom of the shoulder and elbow joints.

**Methods:**

Six degree-of-freedom load cell data along with eight channels of EMG data were recorded during eight tasks (shoulder abduction and adduction, horizontal abduction and adduction, internal rotation and external rotation, and elbow flexion and extension) and used to create feature sets for LDA-based classifiers to distinguish between these eight classes.

**Results:**

Cross-validation yielded functional offline classification accuracies (> 90%) for two of the eight classes using EMG-only, four of the eight classes using load cell-only, and six of the eight classes using a combined feature set with average accuracies of 83, 91, and 92% respectively.

**Conclusions:**

The most common misclassifications were between shoulder adduction and internal rotation followed by shoulder abduction and external rotation. It is unknown whether the strategies used were due to abnormal synergy or other factors. LDA-based pattern recognition may be a viable control option for predicting movement intention and providing a control signal for a wearable exoskeletal assistive device. Future work will need to test the approach in a more complex multi-joint task, specifically one that attempts to tease apart shoulder abduction/external rotation and adduction/internal rotation.

## Background

Nearly 800,000 people in the U.S. and 16 million people worldwide suffer a stroke each year [1]. Of these, an estimated 50% result with chronic hemiparesis [2] and up to 80% may have residual upper-extremity impairments [3]. Commonly these survivors present with abnormal movement patterns referred to as abnormal synergies [4, 5] described as a loss of independent joint control due to coactivation of muscles across multiple joints [6]. Proximal shoulder abduction effort causes involuntary elbow, wrist, and finger flexion, as well as forearm supination proportional to the amount of shoulder effort and is referred to as the flexion synergy [7–9]. In the same manner, shoulder adduction produces involuntary elbow extension, wrist and finger flexion, and forearm pronation and is referred to as the extension synergy. The loss of independent joint control resultant from abnormal synergies is thought to be the result of increased utilization of the contralesional corticoreticulospinal tract [10, 11].

When shoulder effort is reduced, there is a proportional reduction in the expression of loss of independent joint control, enabling access to a greater functional workspace, with full support of the shoulder leading to near maximal reaching range of motion [7, 12]. While targeting this impairment with progressive abduction loading therapy has provided small benefit [13, 14] the complete restoration of movement remains elusive. Therefore, one possible solution to aid these stroke survivors with persistent loss of independent joint control is to support their arm with a wearable exoskeleton. This exoskeleton could provide smart-support possibly leading to long-term improvements in workspace. At a minimum, a wearable device could assist and enable a survivor of stroke by minimizing the effects of abnormal synergy and therefore maximizing their functional work area, better engaging their environment, and/or supporting interventions for their hand, wrist, and elbow. Powered exoskeletons, for both upper- [15] and lower- [16] extremity, are becoming more commonplace and are beginning to emerge as viable sources of rehabilitation and assistance. However, design requirements and feasible control techniques that consider the expression of abnormal synergy/loss of independent joint control have not been established.

The application of wearable robotic technology has found success in individuals with amputation [17] paving the way for potential use in individuals with stroke. Historically, these devices were controlled using simple amplitude-based thresholds, but recently the use of linear discriminant analysis (LDA) based pattern recognition has proven to be both accurate and computationally efficient and enables intuitive control of a greater number of degrees of freedom [17–19]. Although LDA-based pattern recognition is often focused on controlling distal joints, pattern recognition of shoulder motions of healthy controls has been explored for the purposes of application to the population with amputation and has achieved classification accuracies above 90% [20, 21]. 90% is significant, as it has been implicated as a transitional value between high functionality and extremely variable levels of functionality of a myoelectric prosthesis based on the user, classifier, and their interaction [22].

Pattern recognition has been implemented with individuals with stroke with varying degrees of success. Electromyography (EMG) data from the forearm has been used to predict movement with low [23], mixed [24], and high [25] levels of accuracy. Additionally, EMG has been used to predict goal-directed horizontal reaching in both impaired and healthy controls reporting insufficient and sufficient accuracy respectively [26]. Importantly, a limitation to these applications in individuals with stroke was that the participant’s arms were supported, minimizing the expression of the abnormal synergy and potentially inflating classification accuracies compared to what they would be during active shoulder use common in activities of daily living. In fact, classification accuracy for determining an individual’s desire to open their hand is significantly reduced when lifting as little as 25% of their abduction maximum [9, 27]. Advanced offline techniques for correcting synergy-induced classification errors only appreciably improved one subject’s accuracy [9]. Even if these classification errors could be corrected, the effects of abnormal synergy at the elbow, wrist, and fingers (unintentional activation of muscles) would still exist, possibly limiting range of motion or requiring an exoskeleton to mechanically overpower each affected joint. To reduce synergy presentation and achieve success in classifying distal movement intent, abduction support (less required abduction torque generation) is required. With the advancement of wearable robotics, it is feasible to envision a device that could do this. Actively and smartly controlling abduction support would be desirable in hopes of both facilitating recovery and avoiding “slacking” (tendency to over utilize device support leading to increased weakness). Accurate classification of movement intent at the shoulder and perhaps the elbow would be required to realize this goal but has never been demonstrated. Therefore, as a first step, this study aims to determine if LDA-based pattern recognition of shoulder and elbow joints can achieve functionally useable classification accuracies (> 90%) despite the existence of the abnormal synergies in a rigorously controlled and quantitative paradigm.

Sensor fusion from multiple sources has been shown to supplement LDA-based pattern recognition to predict ambulation [28, 29]. The utilization of force and moment load cell data, is a unique and potentially powerful control option available for application of pattern recognition in individuals with stroke that is not available to individuals with amputation; the incorporation of which may augment the accuracy of a solely EMG-based classifier for individuals with stroke. This study investigates how effectively LDA-based pattern recognition techniques applied to load cell, joint torque, EMG, and combined data, classify between maximal isometric torque tasks in eight different directions (shoulder abduction/adduction, shoulder horizontal abduction/adduction, shoulder internal/external rotation, and elbow flexion/extension). It was hypothesized that the combination of load cell and EMG data would result in the largest number of tasks with classification accuracies > 90%.

## Methods

### Participants

Informed consent was obtained from participants to complete the protocol approved by Northwestern’s Institutional Review Board. Thirty-five participants with chronic stroke and upper-extremity Fugl-Meyer assessment (UE-FMA) scores between 10 and 45, classifying their motor impairment as moderate or severe, were recruited to participate in the experiment. Six participants were excluded; two for corrupted or absent data that resulted in less than three useable trials in a given direction, and four for profound external rotation weakness. These four participants were unable to produce external rotation torque with the exception of that which occurred as a secondary torque (i.e. when testing in a different direction). Characteristics from the remaining 29 participants: 21% female, 38% with affected/hemiparetic right arm, average UE-FMA score of 27.4/66 ± 6.4 (15–43), average age of 57.1 ± 9.2 years (37.1–68.7), average time post-stroke 7.2 ± 4.9 years (.98–24.6). Characteristics are presented as percentage or as average value ± standard deviation (minimum value – maximum value).

### Setup and instrumentation

**Fig. 1:**
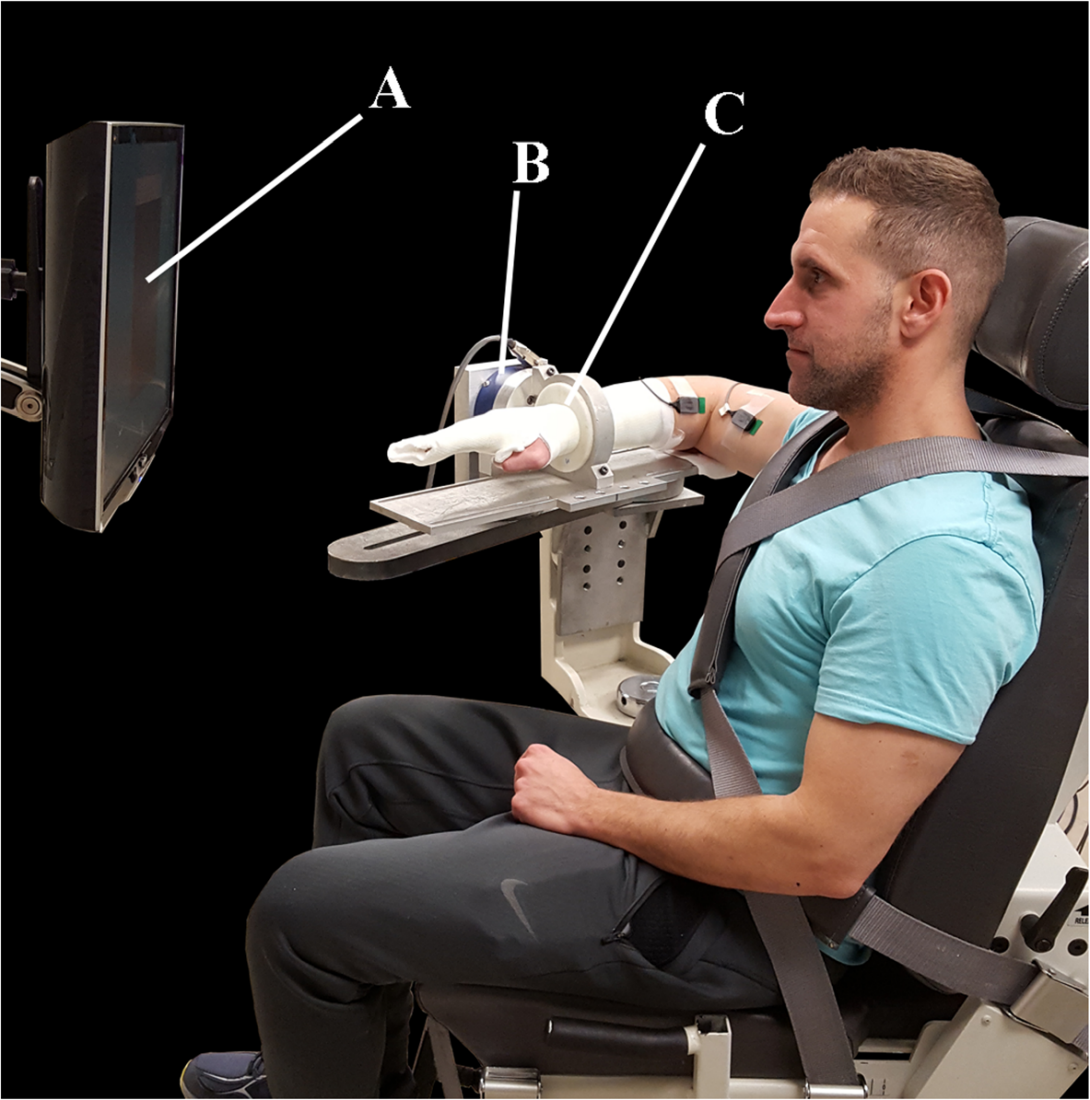
Setup in Biodex chair with participant attached to load cell via rigid cast. Labels identify the following: **a**) Feedback display, **b**) Load cell, **c**) Delrin ring and cast

Participants were seated in a rigid chair (Biodex, Shirley, NY; Model 830–110) and secured with two chest straps and a lap belt to minimize shoulder girdle and torso movement with feet supported by a footrest. Their paretic forearm, wrist, and hand were then casted using fiberglass casting material to provide rigid coupling to a load cell and prevent synergy induced wrist and finger flexion. Using a Delrin ring, the forearm was attached to a 6-degree of freedom (DOF) load cell (JR3 Inc., Woodland, CA, USA; Model 45E15A) that provided instantaneous forces in three orthogonal directions and the moments about each of the three axes enabling the calculation of joint torques at the elbow and shoulder. The custom device was then adjusted to place their paretic arm in a position of approximately 90° of abduction, 45° of horizontal adduction, neutral humeral internal-external rotation, and 90° elbow flexion resulting in the entire arm being in the transverse plane located at shoulder height (Fig. 1). Skin was prepared using a dry scrub pad and alcohol wipe and electrode gel was applied to eight surface-EMG bipolar differential electrodes with 1 cm interelectrode spacing (Delsys, Cambridge, MA, USA; 16 channel Bagnoli) that were attached over the following muscles: anterior deltoid, intermediate deltoid, posterior deltoid, pectoralis major, biceps brachii, triceps long head, triceps lateral head, and brachioradialis. Electrodes were placed by a physical therapist via the use of anatomical landmarks and palpation as prescribed by the book *Anatomical Guide for the Electromyographer* [30]. A ground reference electrode was placed over the acromion. These muscle sites were chosen as they could all be reached by the participants with the intent to mimic feasible self-applied electrode sites for future applications. This choice became a limitation as EMG data from rotator-cuff muscles and latissimus dorsi may have improved differentiation between classes.

### Experimental protocol

**Fig. 2:**
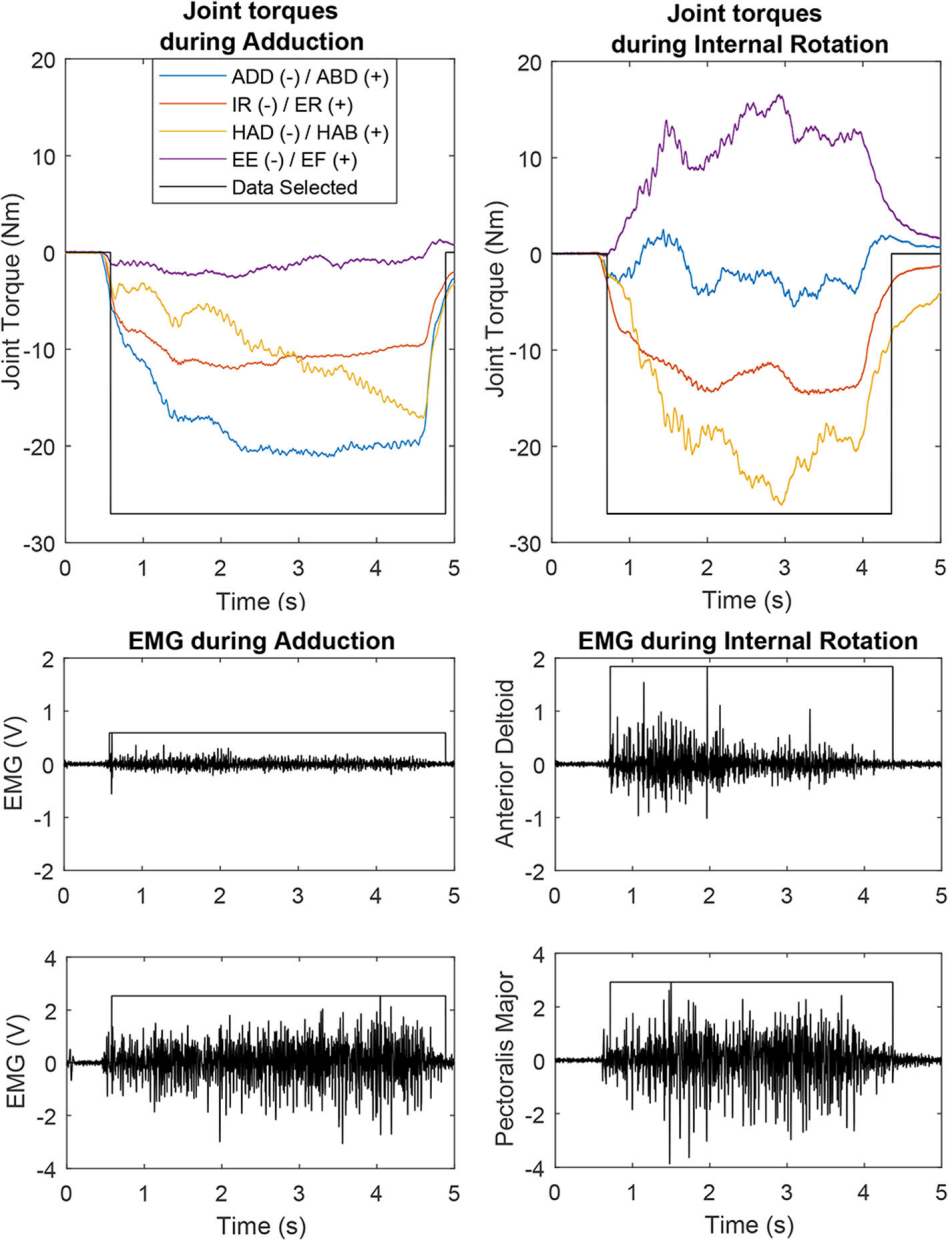
Top) Sample joint torque for one trial of two different shoulder tasks: shoulder adduction and internal rotation, with delineation of which data were selected (> 20% of max in tested direction) for use in the classifier. Bottom) Sample EMG for 2 of the 8 channels: anterior deltoid (used during internal rotation), and pectoralis major (used in adduction and internal rotation). Shoulder Adduction (ADD)/Abduction (ABD); Internal (IR)/External (ER) rotation; Horizontal Adduction (HAD)/Horizontal Abduction (HAB); Elbow Extension (EE)/Elbow Flexion (EF); Newton-meters (Nm); Volts (V); Milliseconds (ms)

Maximum isometric voluntary torques were tested in eight different directions: shoulder abduction (AB) and adduction (AD), horizontal abduction (HAB) and adduction (HAD), internal rotation (IR) and external rotation (ER), and elbow flexion (EF) and extension (EE). Shoulder abduction and adduction torque are defined as the torque generated in the frontal plane around the sagittal axis running through the glenohumeral joint. In this study even though the arm was positioned out of the frontal plane by 45° we still refer to these motions using these terms. Abduction would cause the humerus to rotate cranially while adduction would cause the humerus to rotate caudally, down toward the torso. Horizontal adduction torque would cause rotation around the vertical axis running through the glenohumeral joint in which the humerus would rotate in the transverse plane toward the front of the body and horizontal abduction would rotate the humerus out to the side and then behind the body. Internal rotation torque is similar to what is used in arm-wrestling or overhand throwing and causes rotation of the humerus along the long-axis of the bone. Internal rotation is what allows individuals to reach the small of their back while external rotation allows them to reach behind their head. Torque generated in the testing direction was considered the primary torque while concurrent torques generated in the other directions were labeled as secondary. The order of testing direction was randomized. Each direction was tested a minimum of three and maximum of six trials aiming to satisfy the following criteria: three trial maximum primary torques within 10% of each other with the last trial not being the greatest. Three trials were chosen to allow for adequate data to train and validate each classifier and to allow for some natural variation in task completion without causing fatigue. Verbal directions and visual demonstrations were provided prior to the execution of each task. Real-time visual feedback of torque production in the testing (primary) direction was provided via a large monitor and custom round dial display. The visual feedback offered additional encouragement in addition to auditory encouragement to ensure maximum torque was attained. Trials were 5 s long and recorded at 1 kHz via a data acquisition device (National Instruments, NI-DAQ, Austin, TX, USA). A minimum of one minute of rest was given between trials to ensure adequate recovery time and prevent fatigue. Representative data are presented in Fig. 2.

### Data processing

All data collection, processing, classification, and analyses were accomplished with MATLAB (Release 2012a and 2017a, The MathWorks, Inc., Natick, MA, USA) via custom code. Upon inspection of raw EMG data, a small amount of power line noise was noted, so in addition to the Delsys hardware bandpass filter between 20 to 450 Hz, EMG from each trial was digitally notch filtered between 58 and 62 Hz and subsequent harmonics using a 6th order Butterworth to remove the power line noise. The proceeding analysis was accomplished for both filtered and unfiltered data and the filtered data performed slightly better, but not significantly (1–2%). An in-depth comparison between these datasets was outside the scope of this study. Isometric joint torques were calculated using a series of matrix translations and rotations of the raw six degree-of-freedom load cell data (three forces and three moments) based on limb anthropometrics and relative limb and load cell location and orientation. Maximum voluntary torque values were calculated using a 200 ms moving average. Data were used from the three trials with maximum torque values. Within each trial, automatic segmentation when the primary torque was > 20% of the maximum generated torque for that direction was used for subsequent analysis (classification). Only data when the torque in the primary direction was greater than 20% was used. This somewhat arbitrary cutoff of 20% was used to help ensure that the participant was actually doing what they were tasked to do as well as ensure there was sufficient data to train and validate the classifier for all participants. True maximum strength values are normally achieved for approximately 1 s, so additional data was used to create a richer and lengthier dataset.

### Classification

To evaluate the possibility of predicting user-intent during these shoulder tasks, pattern recognition analyses were performed considering the following signal sources: raw measurements from the load cell only, computed joint torques of the shoulder and elbow only, EMG signals only, and a combination of load cell and EMG sensor sources. The pattern recognition system used in this work is similar to a real-time pattern recognition control system that has been developed to control advanced upper-limb prostheses [31]. The control algorithm contains three basic functions: data windowing, feature extraction, and classification. Windows were formed from 200 ms of data and decisions were made every 25 ms (i.e. 175 ms of overlapping data) maximizing decision density and minimizing delay without significant loss in accuracy [32]. The features used depended on the input signals: for load cell data and computed joint torques, only the mean of each of the channels over each window was used, while for EMG data, the time-domain features proposed by Hudgins [33] were calculated for each of the eight channels. These included mean absolute value, number of zero crossings and slope sign-changes, and waveform length. This resulted in a six-dimensional feature set using load cell, four dimensional set for joint torque data (shoulder abduction/adduction, shoulder horizontal abduction/adduction, shoulder external rotation/internal rotation, and elbow flexion/extension), 32-dimension set for EMG, and 38 for a combined set (6 dimensions from load cell data and 32 from EMG). The extracted features were supplied to an LDA-based classifier. A trial wise leave-one-out-cross-validation was used such that each combination of two trials was used to train a classifier that was then tested against the third, and the accuracies averaged.

## Results

This study takes a focused look at classifying movement patterns at the shoulder and elbow post-stroke following prior work reporting challenges in classifying movements at the forearm, wrist, and hand. Confusion matrices for the load cell and the EMG time-domain feature sets are shown in Tables 1 and 2 respectively. Accuracies above 90% have been shown to be functionally useable while accuracies between 65 and 90% may or may not be, depending on the user, the classifier, and their interaction [22]. Both matrices show error primarily occurring “within synergy”, i.e. between directions that have been implicated in the typical abnormal movement patterns (identified by the major row labels "Flexion" and "Extension" ). Classification errors between abduction and external rotation and adduction and internal rotation are the highest and are most commonly confused for each other.

**Table 1:** Confusion matrix for classifier using the load cell data

Load cell dataset			Predicted Class							
			*EF*	*AB*	*ER*	*HAB*	*EE*	*AD*	*IR*	*HAD*
Actual Class	Flexion	*Elbow Flexion (EF)*	**92**	4	3	1	0	0	0	0
		*Abduction (AB)*	3	**87**	10	0	0	0	0	0
		*External Rotation (ER)*	2	8	**89**	1	0	0	0	0
		*Horizontal Abduction (HAB)*	0	0	2	**97**	0	0	0	0
	Extension	*Elbow Extension (EE)*	0	0	0	0	**98**	1	0	0
		*Adduction (AD)*	0	0	1	0	1	**84**	13	0
		*Internal Rotation (IR)*	0	0	0	0	1	14	**83**	2
		*Horizontal Adduction (HAD)*	0	0	0	0	1	0	2	**96**

Data are averaged across all participants and rounded to nearest whole number. Movements implicated in flexion synergy are in upper/left portion of table while extension synergy movements are in lower/right portion. Bold numbers identify class accuracy while non-bold numbers indicate percent of misclassification. *Flexion synergy*: Elbow flexion (EF), shoulder abduction (AB), external rotation (ER) and horizontal abduction (HAB). *Extension synergy*: elbow extension (EE), shoulder adduction (AD), internal rotation (IR), and horizontal adduction (HAD)

**Table 2:** Confusion matrix for classifier using EMG data

EMG dataset			Predicted Class							
			*EF*	*AB*	*ER*	*HAB*	*EE*	*AD*	*IR*	*HAD*
Actual Class	Flexion	*Elbow Flexion (EF)*	**88**	5	3	2	0	0	1	1
		*Abduction (AB)*	6	**76**	17	1	0	0	0	0
		*External Rotation (ER)*	2	17	**76**	4	0	0	0	0
		*Horizontal Abduction (HAB)*	2	1	6	**91**	0	0	0	0
	Extension	*Elbow Extension (EE)*	0	0	0	0	**89**	5	3	2
		*Adduction (AD)*	0	0	0	0	3	**78**	17	2
		*Internal Rotation (IR)*	0	0	0	0	3	18	**76**	4
		*Horizontal Adduction (HAD)*	0	0	0	0	1	3	6	**90**

Refer to Table 1 text for description. Larger classification errors using EMG alone as compared to other classifiers

A summary of classification accuracies across all participants for each data set is displayed in Table 3. It is clear that the classifier using the load cell dataset outperforms the one using the EMG features dataset and that EMG features add a small improvement when used in conjunction with load cell data, especially with adduction and internal rotation. Abduction, external rotation, adduction, and internal rotation have the lowest average accuracies across all datasets.

**Table 3:** Summary of classification accuracies for each classifier across all participants

	Dataset			
Class	EMG-TD	Torque	Load cell	EMG + LC
EF	88	89	**92**	**94**
AB	76	85	87	**90**
ER	76	87	89	**90**
HAB	**91**	**97**	**97**	**97**
EE	89	**97**	**98**	**97**
AD	78	80	84	87
IR	76	76	83	87
HAD	**90**	**96**	**96**	**98**
Average	83	88	**91**	**92**

EMG-TD refers to EMG time-domain features, Torque to the mean-absolute value (MAV) of torques generated at the shoulder and elbow only, load cell refers to MAV from the raw load cell data, and EMG + LC are the EMG time-domain features and the MAV from the raw load cell data combined together. Bold indicates ≥90% accuracy

**Fig. 3:**
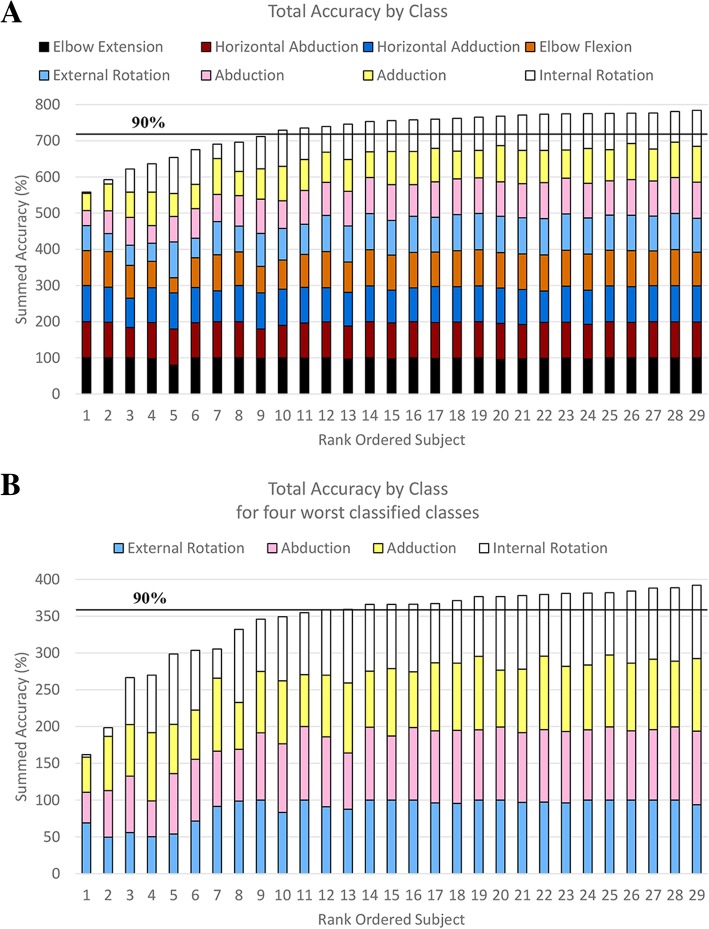
**a**) Stacked bar graph of accuracies from the load cell based classifier for all classes and participants. Classes ordered from most accurate at the bottom to least accurate at the top. **b**) A simpler representation for the four worst classified classes. Participants rank ordered based on total accuracy of the presented classes. Black horizontal line represents a general cutoff for highly functional levels of classification accuracy (90%). Classification accuracy for these four lowest classes range from 50 to 99%, 42 to 95%, 48 to 99%, and 3 to 99% for External rotation, Abduction, Adduction, and Internal rotation respectively

Figure 3a (top) parses out how each class performs using the load cell classifier across all participants. The load cell based classifier generally classifies well (> 90%) for 20 of 29 of the participants. Figure 3b (bottom) shows the same information but only for the four most confused classes. The classification accuracy for these four classes was as low as 50, 48, 42, and 3% for abduction, adduction, external rotation, and internal rotation respectively.

**Fig. 4:**
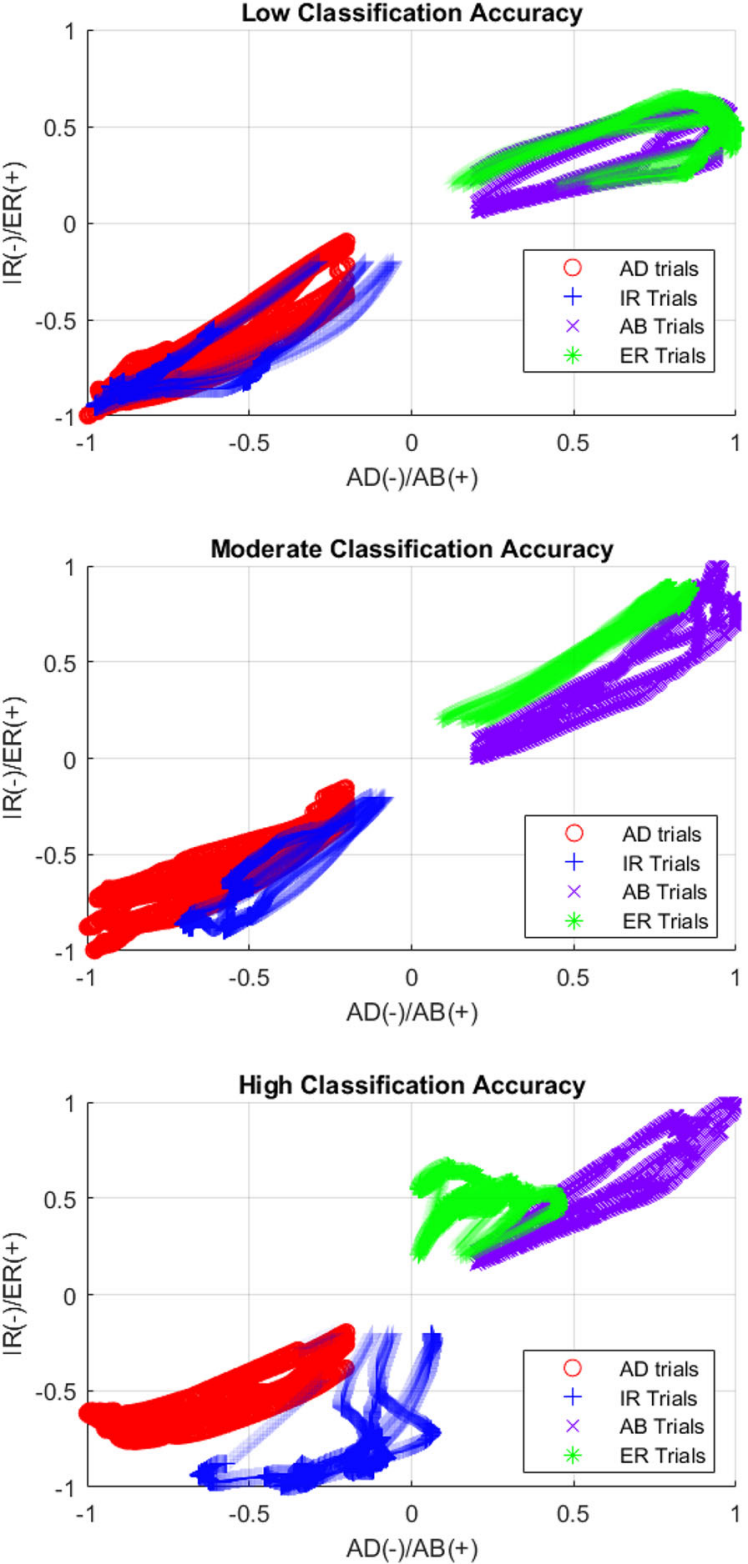
Representative plots of Abduction (AB)/Adduction (AD) vs External rotation (ER)/Internal rotation (IR) normalized torques for 3 participants: low accuracy, moderate accuracy, and high accuracy during all abduction, adduction, internal and external rotation trials. Discrimination between these classes improves as each task is performed in a more unique fashion

In attempt to understand why the classifier was less accurate with these nine participants in these directions, the normalized abduction/adduction joint torque was plotted against the corresponding external/internal rotation joint torque. Trials of representative participants that had low, moderate, and high classification accuracy in these four classes (participants 2, 15, and 29 in Fig. 3), are plotted in Fig. 4. Although this representation does not take into account all data that are used to train and test each classifier, a trend emerges. The participant with the low classification accuracy has the greatest overlap between the torques generated during these different shoulder tasks. It appears that this participant was doing the same or a very similar action for both abduction and external rotation as well as adduction and internal rotation. The plot labeled moderate classification accuracy shows some overlap in the torque generation pattern used to accomplish these single-DOF tasks. Finally, the participant with the highest classification accuracy has the greatest difference in torque patterns in these directions.

**Fig. 5:**
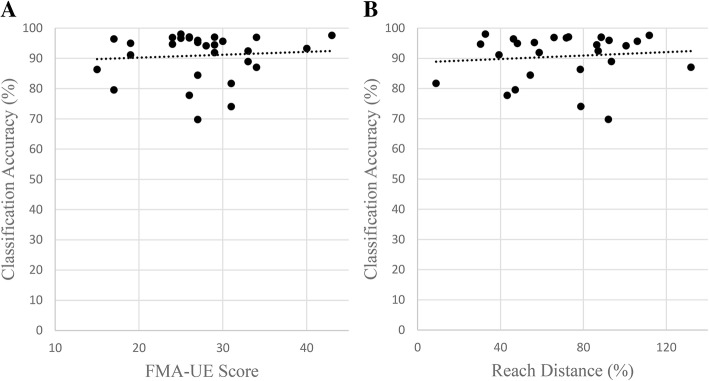
Scatter plots of Classification Accuracy vs UE-FMA (**a**) and Reach area (**b**) for all participants. No significant correlations were found using this data or subsets

A post hoc analysis was completed to explore possible correlation between classification accuracy and synergy presentation, measured clinically with the FMA-UE outcome measure (Fig. 5a) and the laboratory-based measure of reaching distance under limb weight (Fig. 5b) [14]. Spearman rank correlations were calculated for the FMA-UE scores vs accuracy (ρ = − 0.028, *p* = 0.884) as the FMA scores are ordinal data and for reach distance vs accuracy (ρ = 0.12, *p* = 0.554) due to non-bivariate normality. Neither one showed a significant correlation. An additional Spearman rank correlation was tested using a subset of the Fugl-Meyer assessment data: sections I-IV of the assessment that are focused on the shoulder and elbow (ρ = 0.15, *p* = 0.430). Recognizing that our data had a cluster of scores at a plateau near maximal classification accuracy, these three correlations were repeated using only the data from the twelve participants who had the lowest classification accuracies. No correlations were found in these analyses: FMA-UE vs accuracy (ρ = 0.29, *p* = 0.358), reach data vs accuracy (*r* = 0.059, *p* = .856), and FMA-UE sections I-IV (ρ = 0.20, *p* = 0.520). Pearson’s correlation was used for this correlation of reach data as the removal of the plateau made the data bivariate normal.

## Discussion

This is the first time that a pattern recognition analysis has been accomplished on shoulder movements for the population with chronic stroke. The classification accuracies for most participants and classes was greater than 90%, indicating that LDA-based pattern recognition may be a viable control scheme for this population and these tasks. The combination of EMG data with load cell data provided the best classification accuracy averaging 92%, while load cell data alone averaged 91%, and EMG alone averaged 83%. Classification errors (Table 1 and Table 2) occurred within the defined abnormal synergy movement patterns (*flexion synergy*: shoulder abduction, elbow flexion, external rotation, and horizontal shoulder abduction; *extension synergy*: shoulder adduction, elbow extension, internal rotation, and horizontal shoulder adduction) but primarily between adduction and internal rotation and abduction and external rotation.

Table 3 shows that similar trends in error between adduction and internal rotation and abduction and external rotation apply across all datasets. Classifier accuracy using EMG data is much lower when used on its own compared to the other datasets. This may be influenced by the fact that EMG was only recorded from major muscles of the upper extremity. This likely impacted the accuracy of the classifier and could potentially be improved through EMG acquisition from rotator cuff muscles and other involved muscle groups. For example, high classification accuracies (> 92%) have been achieved for shoulder movements within a healthy population using eight channels of EMG over muscles of the back and torso and slightly longer window lengths [20, 21]. Despite the aforementioned limitation, the EMG data does a fair job of classifying and, as seen in the combined data set, offers an improvement especially to the most confused classes, increasing the group average for each class closer to the control scheme goal of > 90%.

The effect of abnormal synergy within the shoulder joint has been described clinically [4, 5] and noted scientifically [6]. However, the results of this work brings question to the validity of using a single-DOF task to quantify this abnormal coupling. In a study using single-DOF tasks, Dewald et al. stated: “the control group exhibited a significant coupling between external rotation and abduction and between internal rotation and adduction that was not present in the nonparetic limb of the hemiparetic group” [6]. This is applicable to these findings as 1) coupling was noted in a control population and 2) was not noted on the non-paretic side of the population with stroke. This suggests that a 1-DOF task may be testing tendency rather than true neurologically mandatory patterns or true ability. The groups exploring the abnormal synergy effect on the elbow, wrist, and fingers have moved away from a 1-DOF in favor of a 2-DOF task, which may come closer to testing true ability rather than general tendency. A similar shift in paradigm to a more complex or less constrained task is necessary for testing pattern recognition within the shoulder, as we cannot confidently say if these classification errors were a function of the task, posture, neural constraints of the population after stroke, or lack of ability of the classifier.

To implement a control scheme in a wearable device it would be ideal to have all necessary data come from sites proximal to and including limb segments that are being assisted. In other words, it is best to keep distal and possibly non-supported limb segments free from sensors, thus it is necessary to know if any sensors are required to be placed on the forearm in order to distinguish between these shoulder tasks. Thus, we tested a classifier which used only shoulder and elbow joint torque data as opposed to using all available load cell data. Classification accuracy of a majority of classes was reduced using this dataset, with the most pronounced loss in discriminating adduction and internal rotation (80 and 76% percent accuracy respectively). This indicates that there may be a pronation/supination component, or something else distal to the elbow occurring during adduction, which was different from what was occurring during internal rotation, enabling improved discrimination. Ultimately, this classifier shows promise in being able to control a device without more distal torque information, but further testing is required to determine if higher levels of discrimination between adduction and internal rotation is possible without it.

For many participants, these classifiers adequately discriminated between abduction/external rotation and adduction/internal rotation, but for others, they did not. One reason these tasks were not classified as accurately for a cohort of participants is that these individuals were accomplishing different tasks similarly. Those supporting the idea that abnormal synergy was affecting task performance might say that these participants are physically unable to produce torque in those directions without also generating torque in unintended (secondary) directions. Meaning that these participants are locked into typical or predictable patterns due to their neurophysiologic adaptation to their stroke. If so, classification accuracy would be generally associated with the severity of expression of abnormal synergy. In an ad hoc exploration, we looked for but did not find, a correlation between the UE-FMA outcome measure and classification accuracy (Fig. 5a). Because this outcome measure has limited resolution, classification accuracies were also compared to reaching distance data available for most of these participants from a recent study [14] but similarly, were not associated (Fig. 5b). The reported reach distance is presented as a percent of excursion attained toward a standardized target near end range of motion under limb weight. This does not indicate that these measures are a poor measure of abnormal joint coupling, but that abnormal coupling within the shoulder may not exist or at least not to the same extent as more distal joints (elbow, wrist, fingers). This may also indicate that an alternative explanation is more likely. It is possible that due to the nature of an isometric task, the posture selected for this study, or of being told to push or pull as hard as they can, these participants used strategies to maximize their torque production that serendipitously or veraciously reflected the previously described constrained abnormal synergy pattern (shoulder adduction and internal rotation or shoulder abduction and external rotation). This suggests that single-DOF isometric torque generation tasks are either not as accurate at quantifying loss of independent joint control as multi-DOF tasks such as reaching dynamics under differing loads [34], or are possibly inadequate altogether. Another alternative is that these participants were not performing the same task consistently during the three analyzed trials, causing increased classification error. Considering each of these possibilities, application of the classifier on a more complex task is warranted to determine accuracies in a task that truly represents impairment such as the ability to move outside of these patterns. While a correlation between classification accuracies and expression of abnormal synergy may emerge, the present data would lead one to hypothesize that adequate classification accuracies are possible.

Other features and other classification techniques were not used or explored in this initial analysis as many participants had adequate classification accuracy and we do not feel the loss of accuracy for the others was due to lack of classifier abilities. Rather we think that these participants completed the task in a different way than the others. Specifically, in an attempt to maximize their torque generation, they may be coupling these directions producing a similar pattern whether attempting to elicit torque in one direction or another. The degree to which this multi-joint pattern observed during the single-joint task represents abnormal synergy in unclear. Future work will need to explore classification accuracies in multi-DOF movements outside of these patterns as well as attempt to automatically detect the onset of expression of abnormal synergy in order to move toward real-time control of a wearable shoulder assistive device during functional movements.

## Conclusion

Here we have demonstrated the possibility to classify user-intent of these eight upper-extremity directions to an adequate level for control for most of the individuals (20 of 29) in this study. For some individuals, the classifier had difficulty discriminating between shoulder adduction and internal rotation and shoulder abduction and external rotation. It is unknown if this is due to manifestation of the negative effects of abnormal synergy, a limitation inherent in the posture chosen, or a volitional strategy to maximize torque production. This warrants the evaluation of a more complex multi-DOF task representing a pattern outside of the abnormal synergy. Evaluation of the LDA-based classifier under these conditions including the use of sensors on rotator cuff muscles may also improve accuracies for the challenging torque combinations of abduction/external rotation and adduction/internal rotation.

Accurate classification of movement intent is necessary for the successful implementation of a sensor-driven actuated exoskeleton. This work provides initial evidence supporting the ability to differentiate shoulder and elbow movements despite previous challenges in differentiating more distal upper extremity actions. This suggests that continued work is warranted to investigate if an LDA-based classifier can be an effective solution for the control of a more proximal assistive device. Such a device would have both assistive and restorative potential.

## Abbreviations

AB: Abduction
AD: Adduction
DOF: Degree of freedom
EE: Elbow extension
EF: Elbow flexion
EMG: Electromyography
ER: External rotation
HAB: Horizontal abduction
HAD: Horizontal adduction
IR: Internal rotation
LDA: Linear discriminant analysis
UE-FMA: Upper-extremity Fugl-Meyer Assessment
